# High-Frequency Deep Brain Stimulation of the Substantia Nigra Pars Reticulata Facilitates Extinction and Prevents Reinstatement of Methamphetamine-Induced Conditioned Place Preference

**DOI:** 10.3389/fphar.2021.705813

**Published:** 2021-06-30

**Authors:** Libo Zhang, Shiqiu Meng, Wenjun Chen, Yun Chen, Enze Huang, Guipeng Zhang, Yisen Liang, Zengbo Ding, Yanxue Xue, Yun Chen, Jie Shi, Yu Shi

**Affiliations:** ^1^ Shenzhen Public Service Platform for Clinical Application of Medical Imaging, Shenzhen Key Laboratory for Drug Addiction and Medication Safety, Department of Ultrasound, Peking University Shenzhen Hospital, Shenzhen, China; ^2^ National Institute on Drug Dependence and Beijing Key Laboratory of Drug Dependence, Peking University, Beijing, China

**Keywords:** deep brain stimulation, substantia nigra pars reticulata, methamphetamine, extinction, relapse

## Abstract

Persistent and stable drug memories lead to a high rate of relapse among addicts. A number of studies have found that intervention in addiction-related memories can effectively prevent relapse. Deep brain stimulation (DBS) exhibits distinct therapeutic effects and advantages in the treatment of neurological and psychiatric disorders. In addition, recent studies have also found that the substantia nigra pars reticulata (SNr) could serve as a promising target in the treatment of addiction. Therefore, the present study aimed to investigate the effect of DBS of the SNr on the reinstatement of drug-seeking behaviors. Electrodes were bilaterally implanted into the SNr of rats before training of methamphetamine-induced conditioned place preference (CPP). High-frequency (HF) or low-frequency (LF) DBS was then applied to the SNr during the drug-free extinction sessions. We found that HF DBS, during the extinction sessions, facilitated extinction of methamphetamine-induced CPP and prevented drug-primed reinstatement, while LF DBS impaired the extinction. Both HF and LF DBS did not affect locomotor activity or induce anxiety-like behaviors of rats. Finally, HF DBS had no effect on the formation of methamphetamine-induced CPP. In conclusion, our results suggest that HF DBS of the SNr could promote extinction and prevent reinstatement of methamphetamine-induced CPP, and the SNr may serve as a potential therapeutic target in the treatment of drug addiction.

## Introduction

Persistent and stable drug memories are considered a major contributor to the intense craving and relapse in drug addiction, which are difficult to eliminate (Hyman and Malenka, 2001; Kauer and Malenka, 2007). Even after extinction, when being re-exposed to drug-associated cues, the original drug memories would be reactivated and cause drug-seeking behaviors, leading to a high relapse rate among addicts (Conklin and Tiffany, 2002; Milton and Everitt, 2012b; Chen et al., 2019b). It has been found that extinction combined with other interventions, such as the retrieval–extinction procedure, can facilitate elimination of drug memories and prevent relapse (He et al., 2011; Xue et al., 2012; Xue et al., 2014; Luo et al., 2015; Liu et al., 2019), which provides a new avenue for the treatment of addiction (Milton and Everitt, 2012a).

Deep brain stimulation (DBS) is an FDA-approved therapy for essential tremor (Schuurman et al., 2000; Opri et al., 2020), Parkinson’s disease (Rosin et al., 2011; Okun, 2012; Katz et al., 2015), idiopathic dystonia (Kleiner-Fisman et al., 2007; Elkaim et al., 2019), and severe obsessive-compulsive disorder (Figee et al., 2013; Wu et al., 2021) and exhibits potential therapeutic effects in the treatment of some other neurological and psychiatric disorders, such as depression (Kennedy et al., 2011; Holtzheimer et al., 2017; Crowell et al., 2019), anorexia nervosa (Lipsman et al., 2013; Lipsman et al., 2017), and addiction (Luigjes et al., 2012; Creed et al., 2015). In addition, unlike pharmacotherapy, DBS has the advantages of adjusting stimulus parameters and starting and stopping stimulation at any time based on the condition of patients, and it also produces minimal side effects when used in clinical application (Kringelbach et al., 2007).

Preclinical and clinical studies have indicated that DBS may be effective in the treatment of cocaine (Creed et al., 2015), morphine (Martinez-Rivera et al., 2016), and heroin (Chen et al., 2019a) addiction. For example, studies have proven that high-frequency (HF) DBS of the nucleus accumbens suppresses seeking behavior and reinstatement of cocaine and methamphetamine (Vassoler et al., 2008; Muller et al., 2013; Vassoler et al., 2013; Batra et al., 2017). However, research has also found that HF DBS of the nucleus accumbens could decrease natural reward-seeking behaviors (Guercio et al., 2015). Meanwhile, it has also been found that DBS can exert distinct effects *via* different stimulus parameters (Schor and Nelson, 2019). Thus, proper targets and parameters of DBS in the treatment of addiction are yet to be identified (Wang et al., 2018).

Substantia nigra pars reticulata (SNr) is a part of the basal ganglia which is involved in various brain functions such as sleep and motivation (Liu et al., 2020; Lai et al., 2021) and diseases including PD (Du et al., 2018; Willard et al., 2019; Sitzia et al., 2020) and seizures (Wicker et al., 2019; Chen et al., 2020). The most dominant neuronal cells in the SNr are GABAergic neurons, and previous studies have found that the SNr serves as a superb DBS target for the treatment of PD-related symptoms (Chastan et al., 2009; Valldeoriola et al., 2019). Evidence also suggests that PD and addiction share certain common mechanisms which involve the striatum (Villalba and Smith, 2013), the major input areas of the SNr (Van Den Berge et al., 2017), making it possible to apply DBS of the SNr to the treatment of addiction. A recent study has also found that GABA neurons in the SNr play important roles in opioid reward and relapse, and activation of SNr GABA neurons decreased heroin-primed reinstatement (Galaj et al., 2020). Thus, the SNr has great potential to be an effective target of addiction treatment.

Here, we investigated the impacts of HF and low-frequency (LF) DBS of the SNr on extinction of methamphetamine-induced conditioned place preference (CPP) and methamphetamine-primed reinstatement in rats. We also examined the effects on locomotor ability, anxiety-like behaviors, and formation of methamphetamine-induced place preference.

## Materials and Methods

### Animals

Male Sprague-Dawley rats (260–280 g), purchased from Beijing Vital River Laboratory Animal Technology Co., Ltd., were housed five per cage prior to the implantation of electrodes. All rats were given access to freely available food and water with a reverse 12/12 h light/dark cycle. All procedures were performed in accordance with the National Institutes of Health’s Guide for the Care and Use of Laboratory Animals and were approved by the Biomedical Ethics Committee for Animal Use and Protection of Peking University.

### Implanting the Stimulating Electrodes

After a period of adaptation, the rats were anesthetized with isoflurane and placed in a stereotaxic apparatus. Stainless steel bipolar electrodes were bilaterally implanted into the SNr at the following coordinates: anterior/posterior, −5.3 mm; medial/lateral, 2.3 mm; and dorsal/ventral, −8.2 mm. Electrodes were secured to the skull with anchoring screws and dental acrylic cement. The rats were housed individually after the surgery and allowed 3–5 days of recovery before behavioral experiments.

### Conditioned Place Preference

The CPP procedure in a three-chamber apparatus was performed using an unbiased, counterbalanced protocol as described previously (Liang et al., 2017).

Baseline preference was assessed by placing the rats in the center chamber of the CPP apparatus and allowing them to explore all three chambers freely for 15 min. Rats that showed a strong unconditioned preference for either of the side chambers (i.e., >540 s) were excluded from the experiments. Then the rats were trained for eight consecutive days with alternating injections of methamphetamine (1 mg/kg, i. p.) or saline (1 ml/kg, i. p.) and were confined to the conditioning chambers for 45 min after each injection before being returned to their home cages. The test for the expression of methamphetamine-induced CPP was identical to the initial baseline preference assessment and was performed on the following day after training. After the establishment of CPP, all rats were divided into sham and HF (or LF) DBS groups in an unbiased random manner.

DBS was continuously delivered for 60 min before the extinction sessions. This duration of stimulation was selected based on previous studies showing that 60 min of DBS is sufficient to produce behavioral changes in rats (Martinez-Rivera et al., 2016; Fakhrieh-Asl et al., 2020). A total of six or nine extinction sessions were performed for HF DBS or LF DBS, respectively, until the rats showed no obvious place preference for either chamber. Similar to the expression test, the rats were allowed to move freely between compartments during each extinction session. On the last day, all of the rats received an injection of methamphetamine (1 mg/kg) without DBS and were tested immediately for CPP. The time spent (in seconds) in the methamphetamine-paired chamber minus the time spent in the saline-paired chamber was calculated as the index of the CPP score.

In the experiment of investigating the effect of HF DBS on CPP formation, rats were divided into sham or HF DBS groups based on the baseline preference before training. During the methamphetamine-pairing trials in training, the rats received 60-min sham or HF DBS in their home cages and were then given an injection of methamphetamine (1 mg/kg) and placed into the drug-paired chamber for 45 min. During the saline-pairing trials, the rats received an injection of saline and were placed into the saline-paired chamber for 45 min. The procedure for the test of expression of methamphetamine-induced CPP was identical to that described above.

### Deep Brain Stimulation

Monophasic square pulses were delivered to the SNr using a current-based stimulator through a cable connected to the implanted electrodes. The stimulation parameters were HF (130 Hz) or LF (20 Hz) pulse frequencies, 150 *μ*A pulse amplitude, and 100 *μ*s pulse width (Martinez-Rivera et al., 2016). In sham DBS experiments, the rats were connected to the external cable but did not receive electrical stimulation.

### Elevated Plus Maze

The elevated plus maze was used to determine anxiety-like behavior as previously described (Xue et al., 2015; Fang et al., 2018). The elevated plus maze consisted of four arms set in a plus-shaped configuration. The apparatus was elevated 70 cm above the floor. The two open arms were 50 cm long and 10 cm wide. The two closed arms were 50 cm long and 10 cm wide with 40-cm-high walls. All rats received a 60-min DBS (sham, HF, or LF) in their home cages, and then each rat was placed in the central zone of the elevated plus maze with its head facing an open arm. The rat was allowed to freely explore the elevated plus maze for 5 min under dim illumination. The number of entries into and time (in seconds) spent on the open arms were recorded.

### Open Field Test

The open field test apparatus consisted of a square arena that was 75 cm long, 75 cm wide, and 40 cm high, which was divided into 25 equal squares on the floor of the arena. All rats received a 60-min DBS (sham, HF, or LF) in their home cages, and then each individual rat was placed in the center of the arena and allowed to freely explore for 5 min. The number of crossings (i.e., entering the adjacent square with all four paws) was considered as the index of locomotor activity.

### Histology

The animals were anesthetized and transcardially perfused with 0.01 M phosphate buffer solution, followed by 4% paraformaldehyde in 0.2 M phosphate buffer. The brain was extracted, post-fixed overnight at 4°C, and cryoprotected in 30% sucrose in 0.2 M phosphate buffer. The cannula placements were confirmed in 25-*μ*m-thick sections using Nissl staining by light microscopy. Rats with misplaced cannulae were excluded from the statistical analysis.

### Statistical Analysis

The paired *t* test was used to compare the baseline and the test of the CPP scores from the methamphetamine conditioning phase. Two-way repeated measures ANOVA was applied to analyze the differences in CPP scores of extinction sessions between the sham and HF/LF DBS groups. The unpaired *t* test was used to compare the differences in CPP scores of methamphetamine conditioning or drug-primed reinstatement between the sham DBS and DBS groups. One-way ANOVA was performed to measure the locomotor activity and anxiety-like behaviors of rats between the sham, LF, and HF DBS groups. Data are shown as mean ± SEM, and the statistical analyses and plotting of the graphs were performed using GraphPad Prism 8 (GraphPad Software, California, United States).

## Results

### High-Frequency Deep Brain Stimulation of the Substantia Nigra Pars Reticulata Facilitated Extinction and Prevented the Reinstatement of Methamphetamine-Induced Conditioned Place Preference

**FIGURE 1:**
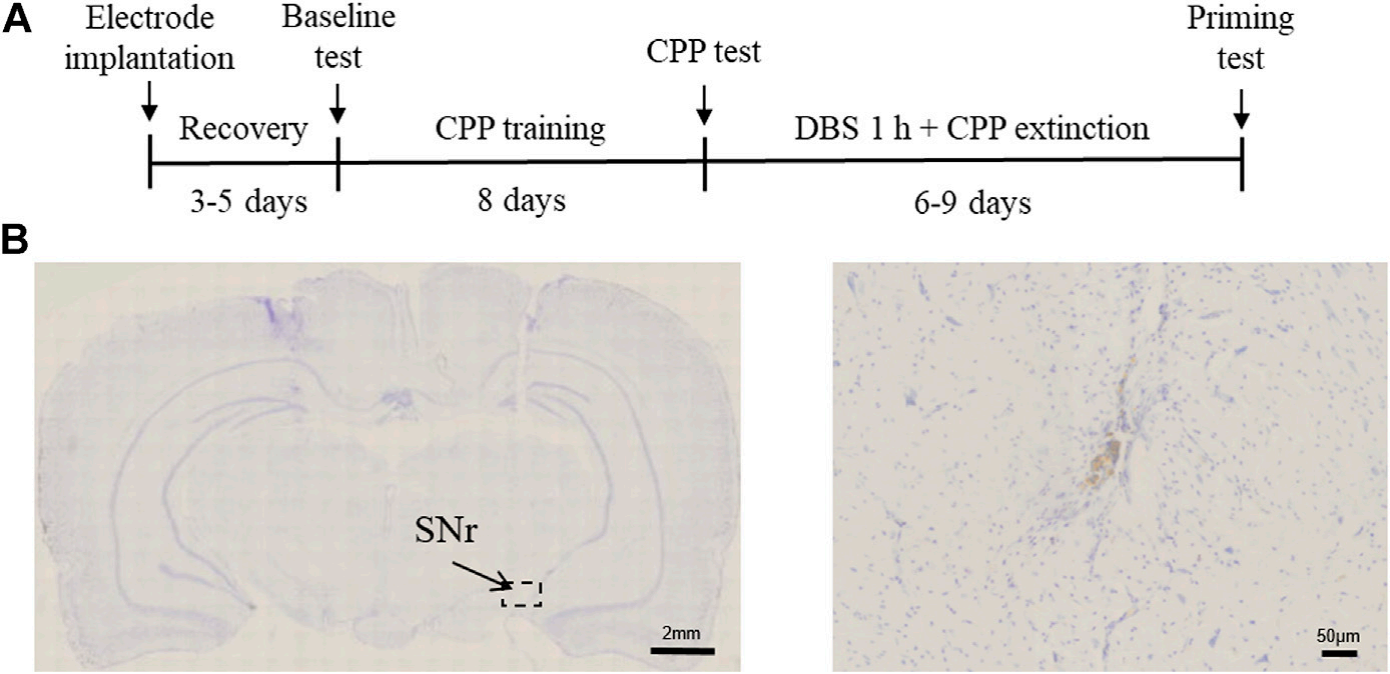
Experimental design and electrode implantation site. **(A)** Experimental timeline. **(B)** Nissl’s staining of the SNr DBS site.

To examine the effect of SNr DBS on extinction and drug-primed reinstatement of methamphetamine-induced CPP, rats were first trained for 8 days with regard to conditioned place preference. After the rats acquired a preference for methamphetamine, DBS was delivered to the SNr for 60 min before each extinction test, and at the end of extinction, an injection of methamphetamine was given to evaluate the reinstatement of drug-seeking behavior (Figures 1A,B). Rats with misplacement of electrodes were excluded from the study.

**FIGURE 2:**
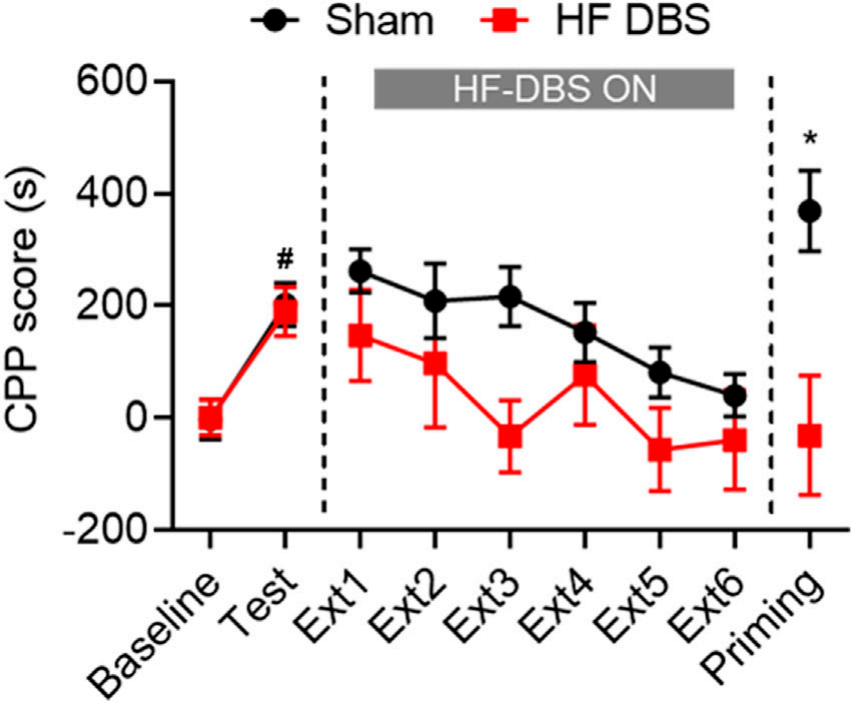
HF DBS of the SNr facilitated extinction and prevented the reinstatement of methamphetamine-induced CPP. Methamphetamine (1 mg/kg) induced a significant preference for the drug-paired side in both groups, and sham DBS or HF DBS (130 Hz, 150 *μ*A, 100 *μ*s) was then applied to the SNr during each of the drug-free extinction sessions (15 min). HF DBS caused a significant decrease in CPP scores compared with the sham DBS. After full extinction, a priming injection of methamphetamine (1 mg/kg) was given to the sham DBS and HF DBS groups, and only the sham DBS group, but not the HF DBS group, showed reinstatement of drug-seeking behavior. Data are shown as mean ± SEM. ^#^ *p* < 0.05 compared with baseline (sham and HF DBS), *p < 0.05 compared with HF DBS. Sham DBS: *n* = 8; HF DBS: *n* = 8.

As shown in Figure 2, two groups of rats exhibited significant preference for the drug-paired side after methamphetamine conditioning (paired *t* test: Sham DBS: *t*_7_ = 3.308, *p* < 0.05; HF DBS: *t*_7_ = 2.868, *p* < 0.05). Sham or HF DBS was then delivered to the SNr during the drug-free extinction phase, and a two-way repeated measures ANOVA showed overall significant differences in the CPP scores across DBS (F_(1, 14)_ = 5.122, *p* < 0.05) and extinction sessions (F_(3.112, 43.57)_ = 3.089, *p* < 0.05) but not DBS × extinction session interactions (F_(5, 70)_ = 0.4994, *p* = 0.7756), which suggests that HF DBS of the SNr facilitated the extinction of methamphetamine-seeking behavior.

Following the last extinction test, all rats received a priming injection of methamphetamine and were tested for reinstatement of drug-seeking behavior. Rats in the HF DBS group showed no significant preference for the methamphetamine-paired side, while those in the sham DBS group exhibited a dramatic increase in CPP scores compared with the HF DBS group (unpaired *t* test, *t*_14_ = 2.178, *p* < 0.05). Therefore, HF DBS of the SNr blocked the methamphetamine-primed reinstatement of the extinguished drug-seeking behavior.

### Low-Frequency Deep Brain Stimulation of the Substantia Nigra Pars Reticulata Impaired the Extinction of Methamphetamine-Induced Conditioned Place Preference and Had No Effect on Methamphetamine-Primed Reinstatement

**FIGURE 3:**
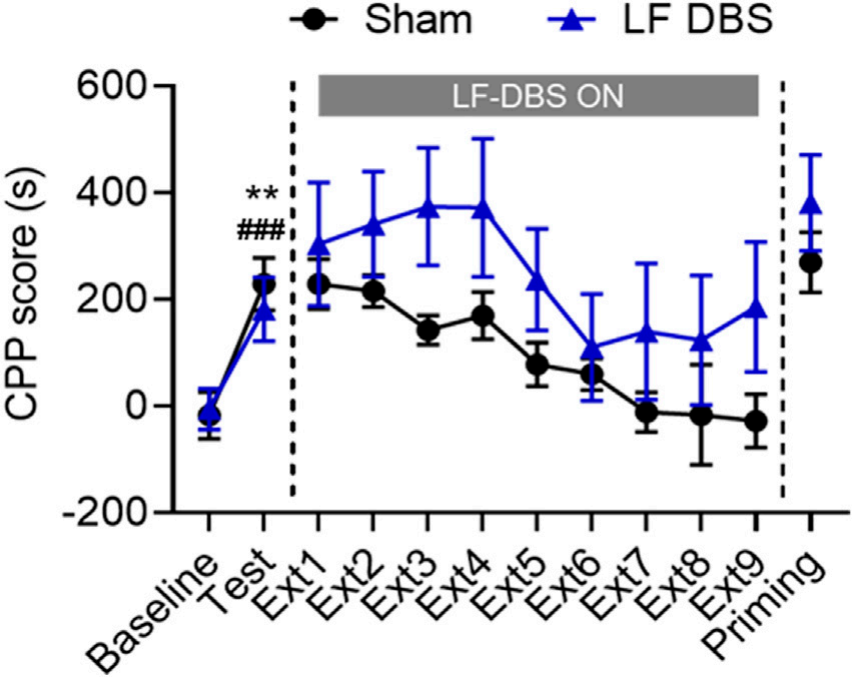
LF DBS of the SNr impaired the methamphetamine-induced CPP extinction and had no effect on methamphetamine-primed reinstatement. After methamphetamine conditioning (1 mg/kg), sham DBS or LF DBS (20 Hz, 150 *μ*A, 100 *μ*s) was delivered into the SNr during each of the drug-free extinction sessions (15 min). LF DBS significantly impaired the extinction sessions compared with the sham DBS. A priming injection of methamphetamine (1 mg/kg) was given to the sham DBS and LF DBS groups, and both groups of rats exhibited significant drug-seeking behaviors. Data are shown as mean ± SEM. **p < 0.01 (sham DBS) and ^###^ *p* < 0.0001 compared with baseline (LF DBS). Sham DBS: *n* = 7; LF DBS: *n* = 6.

Since studies have proven that LF DBS generally has different effects compared with HF DBS on the excitability of the stimulated brain region (Kringelbach et al., 2007; Wang et al., 2018), we examined the effects of LF DBS on the extinction of methamphetamine-induced CPP. As shown in Figure 3, after conditioning, the rats showed an overall preference for the drug-paired side (paired *t* test: Sham DBS: *t*_6_ = 4.003, *p* < 0.01; LF DBS: *t*_5_ = 11.17, *p* < 0.0001). The two groups of rats both underwent extinction until the methamphetamine-seeking behavior of the sham DBS rats was fully extinguished. A two-way repeated measures ANOVA revealed that rats that received LF DBS of the SNr before the extinction sessions exhibited overall significantly higher CPP scores during extinction across DBS (F_(1, 11)_ = 6.473, *p* < 0.05) and extinction sessions (F_(3.737, 41.11)_ = 5.041, *p* < 0.01) but not DBS × extinction session interactions (F_(8, 88)_ = 0.5160, *p* = 0.8415) compared with the sham DBS group. Then all rats received an injection of methamphetamine for the drug-priming test, and the unpaired *t* test revealed no significant difference in the CPP scores between the sham DBS and LF DBS groups (*t*_11_ = 1.374, *p* = 0.1969). Thus, LF DBS of the SNr impaired the extinction of methamphetamine-induced CPP and produced no effect on reinstatement.

### Deep Brain Stimulation of the Substantia Nigra Pars Reticulata Did Not Affect Locomotor Activity and Anxiety-Like Behavior

**FIGURE 4:**
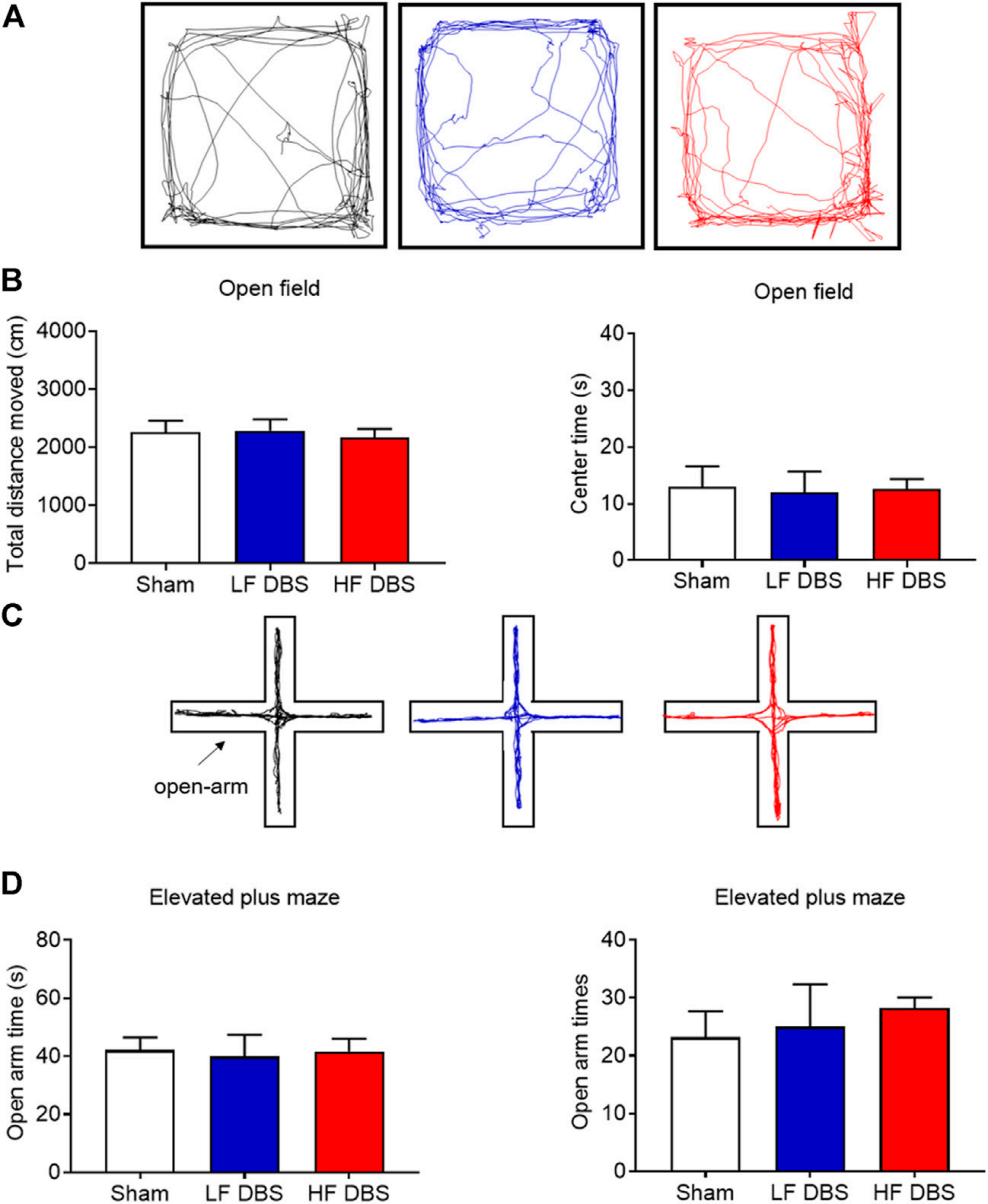
DBS of the SNr did not affect locomotor activity and anxiety-like behavior. **(A)** Representative activity traces of sham, LF, and HF DBS groups in the open field test. **(B)** Total distance traveled and time in the central zone in the open field test. **(C)** Representative activity traces of sham, LF, and HF DBS rats in the elevated plus maze test. **(D)** Time and entries in the open arms of the elevated plus maze test. Data are shown as mean ± SEM. *n* = 6 for all groups.

To rule out the possibility that SNr DBS may have adverse effects on locomotor activity and induce anxiety-like behavior, we used the open field test and the elevated plus maze test to measure these behaviors. HF DBS or LF DBS was delivered into the SNr for 60 min before the tests. One-way ANOVA showed that there was no significant difference in the distance traveled (F_(2, 15)_ = 0.1107, *p* = 0.8959) and the time in the central zone (F_(2, 15)_ = 0.02149, *p* = 0.9788) between the HF DBS, LF DBS, and sham DBS groups in the open field test (Figures 4A,B). Also, no significant difference was found in the open-arm time (F_(2, 15)_ = 0.04105, *p* = 0.9599) and entries (F_(2, 15)_ = 0.2453, *p* = 0.7856) between the HF DBS, LF DBS, and sham DBS groups in the elevated plus maze test (Figures 4C,D). Therefore, SNr DBS had no effect on locomotor activity and did not induce anxiety-like behavior in rats.

### High-Frequency Deep Brain Stimulation of the Substantia Nigra Pars Reticulata Had No Effect on the Formation of Methamphetamine-Induced Place Preference

**FIGURE 5:**
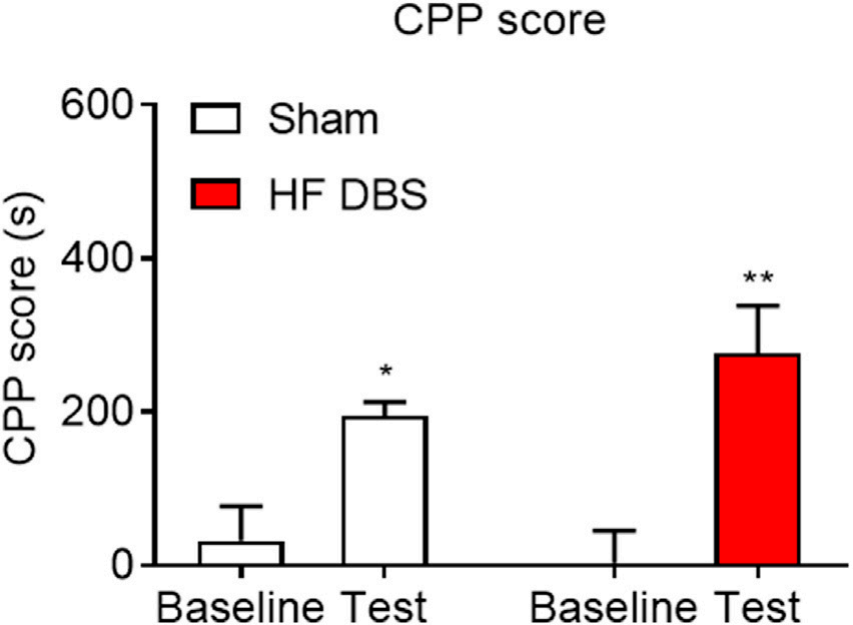
HF DBS of the SNr had no effect on the formation of methamphetamine-induced place preference. Both sham DBS and HF DBS groups exhibited a significant preference for the drug-paired side after the methamphetamine conditioning (1 mg/kg), while no difference was found in the CPP scores between the two groups. Data are shown as mean ± SEM. *p < 0.05 (sham DBS) and **p < 0.01 (HF DBS) compared with baseline. Sham DBS: *n* = 8; HF DBS: *n* = 8.

Finally, we investigated the effect of HF DBS of the SNr on the rewarding effects of methamphetamine. As shown in Figure 5, rats received HF DBS of the SNr before the CPP training, and the paired *t* test showed that both sham (*t*_7_ = 3.494, *p* < 0.05) and HF (*t*_7_ = 4.859, *p* < 0.01) DBS groups formed a significant preference for the drug-paired side, and there was no significant difference in the CPP scores between the two groups (unpaired *t* test, *t*_14_ = 1.272, *p* = 0.2241), indicating that HF DBS of the SNr in the conditioning phase had no effect on the rewarding effects of methamphetamine.

## Discussion

Our data demonstrated that HF and LF DBS of the SNr produced distinct effects on the extinction of methamphetamine-induced CPP. HF DBS of the SNr facilitated the extinction of methamphetamine-induced CPP and blocked drug-primed reinstatement, while LF DBS suppressed extinction. It is worth noting that HF DBS of the SNr did not affect the reinforcing properties of methamphetamine. These findings suggest that the SNr could be a potential DBS target for the treatment of addiction, although proper stimulation parameters and phases need to be chosen.

The SNr is the ventral part of the substantia nigra. Recent evidence has implied that abnormalities of the substantia nigra are involved in the pathophysiology of addiction (Sharpe et al., 2014; Cassidy et al., 2020), and acute methamphetamine administration could induce neuronal death in the substantia nigra (Sabrini et al., 2020). The substantia nigra also plays a crucial role in the relapse to drug seeking (Hyman et al., 2006; Madsen et al., 2012; Pelloux et al., 2018). However, there is still a lack of sufficient evidence on the exact role of the SNr in addiction, and whether intervention in the SNr can be applied in addiction treatment needs further verification.

On the other hand, the SNr is the convergence region of the striatal output pathways, which comprises striatonigral neurons in the direct pathway and striatopallidal neurons in the indirect pathway (Deniau et al., 2007; Phillips et al., 2020). Evidence suggests that the D1-expressing medium spiny neurons of the direct pathway in the striatum project to the SNr GABA neurons and exhibit D1-mediated presynaptic facilitation (Chuhma et al., 2011). Numerous studies have confirmed that the direct pathway is crucial to drug-seeking behaviors (Cui et al., 2014; Volkow and Morales, 2015; Yager et al., 2019; Salery et al., 2020), and inhibition of the activity of striatal neurons in the direct pathway could suppress cue-induced cocaine-seeking behaviors without affecting the formation of cocaine addiction (Yager et al., 2019). Thus, modulating the activity of the SNr may regulate addiction by affecting striatum activity. Studies also found that HF DBS of the SNr produced negative changes in the cerebral blood volume (CBV) in the striatum, and it also evoked positive CBV changes in multiple basal ganglia nuclei as well as the zona incerta and the ventral tegmental area (Van Den Berge et al., 2017), while existing evidence proved that these brain regions play a crucial role in addiction (Hikida et al., 2010; Mahler et al., 2014; Shen et al., 2014). Therefore, electrical stimulation of the SNr may affect addiction by modulating the neural activity of related brain regions.

In the present study, we attempted to investigate the beneficial effects of DBS of the SNr in the treatment of methamphetamine addiction by using the free access CPP extinction paradigm in rats. The stimulation parameters used in previous studies are mainly LF (10–40 Hz) and HF (100–400 Hz) stimulation, while the medium-frequency (40–60 Hz) stimulation has almost no effect on modulating the functional connectivity of the SNr (Creed et al., 2015; Martinez-Rivera et al., 2016; Van Den Berge et al., 2017; Fakhrieh-Asl et al., 2020). Our results indicated that HF DBS of the SNr promoted extinction and subsequently blocked the drug-primed reinstatement. However, we also found that LF DBS of the SNr suppressed the extinction. Besides, HF DBS of the SNr had no effect on the development of methamphetamine-induced CPP. These results suggest that the stages of addiction and the stimulation parameters should be considered when using SNr DBS in the treatment of addiction. On the other hand, DBS has the ability to modulate the synaptic plasticity, which may also contribute to strengthening the extinction memory and suppressing the subsequent reinstatement of drug-seeking behaviors (Kauer and Malenka, 2007; Creed et al., 2015; Lee et al., 2016; Ni et al., 2018). The mechanism of DBS that promotes extinction may be that HF DBS causes long-term potentiation of the SNr and leads to a decrease in the activity of the dorsal striatum, which plays a critical role in the extinction of the addiction memory, in line with the previous findings (Martinez-Rivera et al., 2016; Yager et al., 2019).

Despite the efficacy of DBS in the treatment of a variety of diseases, the underlying mechanisms of these effects remain unclear. Indeed, it is a limitation of the present study that we did not investigate the mechanisms of the effects of DBS of the SNr on extinction and reinstatement of methamphetamine-induced CPP. Studies have found that DBS could enhance the transmission from the stimulation target and activate surrounding fiber pathways simultaneously, leading to a complex pattern of excitatory and inhibitory effects (Miocinovic et al., 2013). Furthermore, consistent with our findings, different frequencies of DBS could produce distinct effects. Acute LF DBS of the nucleus accumbens combined with the dopamine D1 receptor antagonist SCH23390 effectively abolishes the behavioral sensitization of cocaine (Creed et al., 2015). LF DBS of the dorsal ventral striatum strengthens the morphine extinction memory, whereas HF DBS of the dorsal ventral striatum impairs extinction training and the subsequent extinction memory (Martinez-Rivera et al., 2016). Additionally, HF DBS of the OFC prevents the development of morphine place preference and blocks the drug-primed reinstatement of morphine-seeking behavior (Fakhrieh-Asl et al., 2020).

In conclusion, we have found that HF DBS of the SNr facilitated the extinction of methamphetamine-induced CPP and blocked methamphetamine-primed reinstatement, while LF DBS of the SNr impaired extinction. Meanwhile, HF DBS of the SNr neither affected locomotor activity nor caused anxiety-like behaviors. Moreover, it had no effect on the formation of methamphetamine-induced CPP. Our findings may provide potential targets and options for the future clinical application of DBS in the treatment of addiction.

## Data Availability

The raw data supporting the conclusion of this article will be made available by the authors, without undue reservation.
